# Impact of abnormal cesarean section scar formation on quality of life and postpartum depression: A cross‐sectional study in Japan

**DOI:** 10.1002/ijgo.70469

**Published:** 2025-08-14

**Authors:** Yosuke Sugita, Hiroaki Komatsu, Chisato Kodera, Daichi Urushiyama, Naosuke Enomoto, Sumito Nagasaki, Tamami Odai, Tokumasa Suemitsu, Yohei Onodera, Yoshihiko Hosokawa, Rei Ogawa

**Affiliations:** ^1^ Japanese Red Cross Tokyo Katsushika Perinatal Center Tokyo Japan; ^2^ Department of Obstetrics and Gynecology Tottori University School of Medicine Tottori Japan; ^3^ Department of Obstetrics and Gynecology, School of Medical Sciences Kyushu University Fukuoka Japan; ^4^ Department of Obstetrics and Gynecology, Faculty of Medicine Fukuoka University Fukuoka Japan; ^5^ Department of Obstetrics and Gynecology Mie Chuo Medical Center Mie Japan; ^6^ Department of Obstetrics and Gynecology Toho University Omori Medical Center Tokyo Japan; ^7^ Department of Women's Health Institute of Science Tokyo Tokyo Japan; ^8^ Department of Obstetrics and Gynecology The Jikei University School of Medicine Tokyo Japan; ^9^ Department of Obstetrics and Gynecology Akita University Graduate School of Medicine Akita Japan; ^10^ Department of Obstetrics and Gynecology Miyazaki Prefectural Miyazaki Hospital Miyazaki Japan; ^11^ Department of Plastic, Reconstructive and Aesthetic Surgery Nippon Medical School Hospital Tokyo Japan

**Keywords:** abnormal scar, cesarean section, hypertrophic scar, keloid, postpartum depression, quality of life

## Abstract

**Objective:**

To determine the prevalence of abnormal scar formation after cesarean section and evaluate its effects on quality of life (QoL) and postpartum depression risk.

**Methods:**

A nationwide cross‐sectional study of 991 women aged 18–45 years at 6–18 months post‐cesarean section was conducted in Japan. Participants completed validated measures of scar assessment (Dermatology Life Quality Index [DLQI]) and postpartum depression risk (Edinburgh Postnatal Depression Scale [EPDS]).

**Results:**

Among participants, 66.0% Abnormal scarring has been reported to occur more frequently in Asian and Black populations than in White populations, consistent with previous epidemiological studies. (654/991) developed abnormal scars (64.4% hypertrophic, 1.6% keloid). The abnormal scar group showed significantly higher DLQI scores, indicating worse QoL (median 1, interquartile range 0–2 vs median 0, interquartile range 0–1; *P* < 0.001). Women with keloid formation showed elevated EPDS scores, indicating higher depression risk.

**Conclusion:**

Abnormal scar formation is highly prevalent, abnormal scars impair maternal quality of life, and keloid formation is linked to an increased risk of postpartum depression.

## 1 INTRODUCTION

The global cesarean section (CS) rate continues to rise, with projections indicating an increase from 21.1% in 2018 to 28.5% by 2030, reaching up to 50% in some regions.^1^, ^2^ Japan is no exception, with the CS rate reaching 30% in 2023.^3^, ^4^ This increase has raised concerns about the occurrence of abnormal scars and their impact on mental health and quality of life (QoL). However, the importance of preventing abnormal scars is not yet fully recognized.

The primary types of abnormal scars include keloids and hypertrophic scars. Hypertrophic scars are defined as raised scar tissue confined within the wound boundaries, whereas keloids extend beyond the wound boundaries into surrounding normal skin.^5^ These scars not only present esthetic issues but also cause symptoms such as pruritus, pain, and contractures, significantly increasing the physical and psychological burden on patients.^6^, ^7^, ^8^ The CS incision site is subjected to high tension and estrogen exposure, which increases the risk of abnormal scar formation.^9^ Abnormal scarring has been reported to occur more frequently in Asian and African American populations compared with Caucasians.^10^ Previous studies have reported the prevalence of abnormal scarring after CS to be between 31% and 41%.^11^, ^12^ However, these studies were limited by their small sample sizes and, to date, no large‐scale cross‐sectional study has been conducted globally to assess the true prevalence of this complication.

It has been suggested that CS is associated with decreased QoL and an increased risk of postpartum depression (PPD) compared with vaginal delivery; however, the impact of abnormal scar formation on these factors has not been sufficiently investigated. PPD is a serious condition that affects 10%–20% of women worldwide, with approximately 10%–15% of postpartum women in Japan experiencing it.^13^, ^14^ PPD is associated with an increased risk of maternal suicide, impaired mother‐infant bonding, and long‐term problems in children.^15^, ^16^

The present study aimed to investigate the relationship between post‐CS abnormal scar formation and maternal outcomes, hypothesizing that abnormal scarring would significantly impact both QoL and PPD risk.

## 2 MATERIALS AND METHODS

This nationwide cross‐sectional study analyzed data from a web‐based survey originally conducted by Johnson & Johnson K.K. The survey was administered by Rakuten Insight, Inc.^17^ across all 47 Japanese prefectures from January 17 to 23, 2023, targeting women who had undergone CS between July 2021 and July 2022. Inclusion criteria specified women who were 6–18 months post‐CS, ensuring adequate time for scar maturation while minimizing recall bias. Sample size calculation employed Peduzzi's method^18^ (*n* = 10*k*/*p*), where *k* represents the number of predictor variables included in the model, and *p* represents the estimated proportion of participants with the outcome of interest, incorporating 10 predictor variables and an estimated abnormal scar incidence of 0.2, yielding a minimum requirement of 500 participants. Our sample size of 991 participants represents a robust nationwide sample with representation from all 47 prefectures in Japan, providing adequate power for detecting clinically meaningful differences and allowing for robust subgroup analyses, particularly for the rare keloid group. To ensure robust subgroup analyses, particularly for the keloid group (estimated 1% incidence), and account for potential data quality issues, we targeted 1000 participants.

Our data collection protocol incorporated multiple quality assurance measures including automated system checks for response completeness, validation of gestational age entries (22–43 weeks), detection and removal of pattern responses, and geographic representation verification. Responses with implausible or contradictory clinical information were excluded from the analysis to maintain data integrity. Our data collection protocol incorporated multiple quality assurance measures. Initial data quality screening was conducted by Rakuten Insight, Inc. during the collection phase, including automated system checks for response completeness and validation of gestational age entries (22–43 weeks). Subsequently, the first author (Y.S.) conducted additional quality assessments with specific exclusion criteria: (1) implausible clinical information such as delivery dates that significantly deviated from expected delivery dates, and (2) pattern responses where participants selected only extreme values (all ones or all fours) across all questionnaire items, indicating potential inattentive responding. Geographic representation verification was also performed to ensure nationwide coverage. Despite these precautions, as with any internet‐based survey, there remains a possibility that some responses from individuals outside the target population may have been included. This limitation was addressed through rigorous data cleaning procedures and sensitivity analyses to confirm the robustness of our findings. The final analysis included 991 valid responses from 1198 eligible participants, representing a robust 82.7% quality‐assured response rate.

**FIGURE 1:**
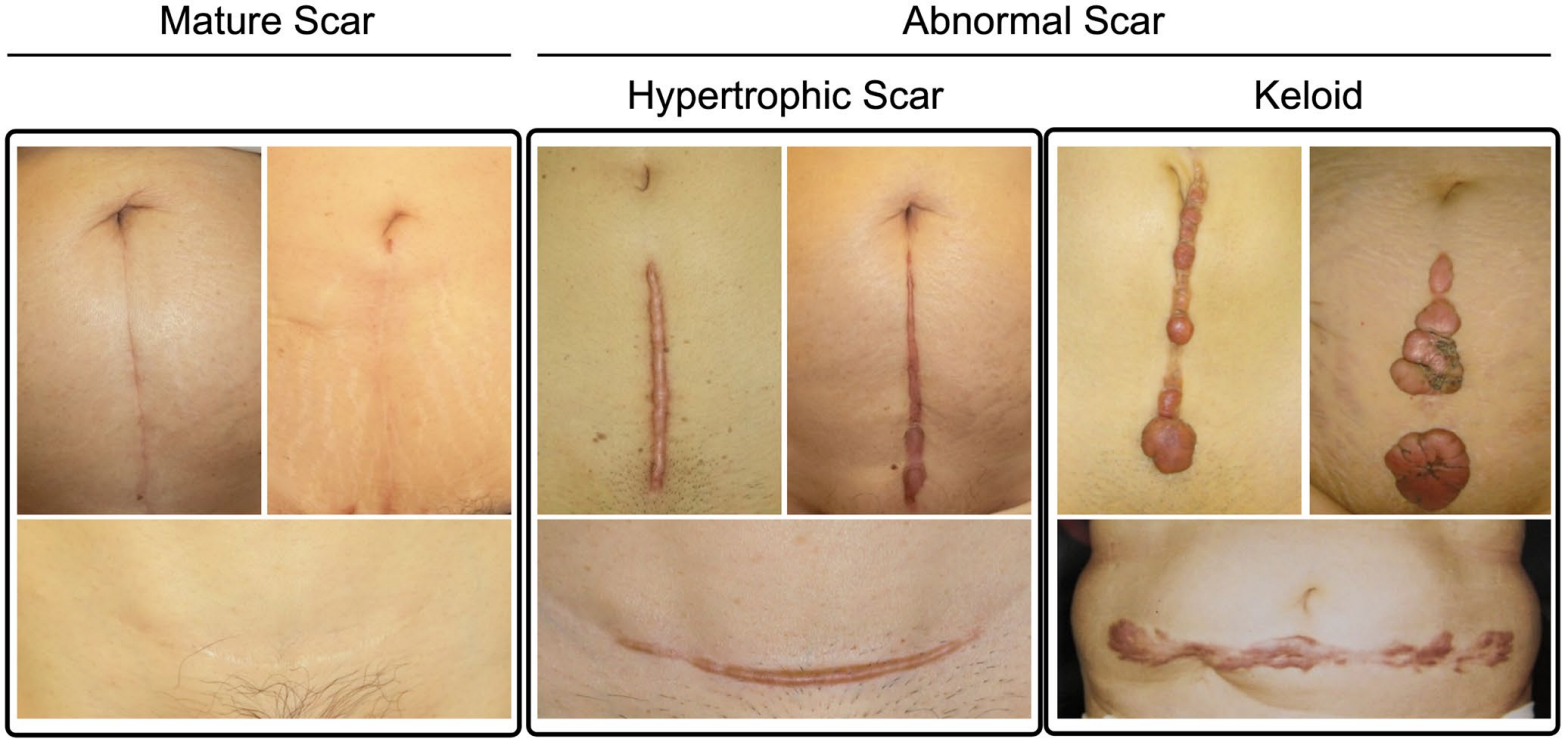
Representative images of cesarean section scars used for self‐assessment. Representative images of mature scars (left panel), hypertrophic scars (middle panel), and keloids (right panel) used in the survey for participant self‐assessment. Participants selected the image that most closely resembled their cesarean section scar. These reference images were carefully selected from typical cases by a plastic surgeon and were photographed with patient consent.

For scar assessment, participants classified their scars using standardized photographic references of mature, hypertrophic, and keloid scars (Figure 1). These reference images were carefully selected from typical cases by a plastic surgeon and were photographed with the previous patients' consent. We acknowledge that this self‐assessment approach represents a significant methodologic limitation, as the JSW Scar Scale 2015 criteria^19^ and established clinical classification systems are specifically designed for physician evaluation, which can assess tactile characteristics such as thickness, texture, firmness, and sensation that are not detectable through photographic assessment alone. Although self‐assessment using photographic references has been employed in large‐scale epidemiologic studies where individual clinical examination is not feasible, no validation studies have specifically compared this approach with the reference standard physician assessment for CS scars. This methodologic compromise was necessary to enable nationwide data collection across all 47 Japanese prefectures, but represents an important limitation that may affect the accuracy of scar classification in our study. QoL was evaluated using the validated Japanese version of the Dermatology Life Quality Index (DLQI),^20^ which assesses symptoms and emotions, daily activities, leisure, work and school, personal relationships, and treatment burden.^8^, ^21^, ^22^ The DLQI is a 10‐item questionnaire with scores that range from 0 to 30, where higher scores indicate greater impairment in QoL. Score interpretation follows established guidelines: 0–1 indicates no effect on QoL, 2–5 indicates small effect, 6–10 indicates moderate effect, 11–20 indicates very large effect, and 21–30 indicates extremely large effect on patient's life. The DLQI assesses six domains: symptoms and emotions, daily activities, leisure, work and school, personal relationships, and treatment burden. PPD screening used the Japanese version of the Edinburgh Postnatal Depression Scale (EPDS),^23^ validated for use up to 1 year postpartum.^24^, ^25^ The EPDS is a 10‐item questionnaire with scores ranging from 0 to 30, where higher scores indicate greater risk of PPD, with scores of 9 or greater indicating a clinically significant depression risk. Depression was defined as persistent feelings of sadness, hopelessness, and loss of interest lasting at least 2 weeks. Anxiety was characterized by excessive worry, fear, and physical symptoms such as restlessness or fatigue. Anhedonia refers to the inability to experience pleasure in activities once found enjoyable. The EPDS evaluates these constructs through validated subscales: anhedonia (items 1, 2), anxiety (items 3, 4, 5), and depression (items 7, 8, 9),^26^ allowing differentiation between these related but distinct psychological states. All 10 items of the DLQI and all 10 items of the EPDS were administered to participants. No items were excluded from the questionnaires or analysis. Domain‐specific analyses were conducted for all six DLQI domains and all three EPDS subscales.

Statistical analysis was performed using R version 4.4.1. We conducted descriptive statistics for demographic and clinical characteristics and employed Mann–Whitney *U* tests for between‐group comparisons of non‐normally distributed DLQI and EPDS scores. Multiple logistic regression analysis was used to identify risk factors, with subgroup analyses focusing on keloid formation. Sensitivity analyses were performed to validate primary findings. Statistical significance was set at a *P* value less than 0.05, with Bonferroni corrections applied for multiple comparisons.

To ensure scientific integrity and mitigate potential bias, data analysis was conducted independently by academic authors without direct involvement from the industry‐affiliated co‐author. Statistical methods and interpretation of results were reviewed by all authors.

The study was approved by the Ethics Committee of the Tokyo Katsushika Red Cross Maternal and Child Medical Center (Approval No. 2303). All participants provided electronic informed consent, acknowledging that survey results might be used for academic research purposes.

## 3 RESULTS

The study achieved comprehensive geographic representation across all 47 Japanese prefectures. Participant demographics and clinical characteristics demonstrated appropriate distribution across age groups, parity, and surgical indications (Table 1).

**TABLE 1:** Demographic and clinical characteristics of cesarean section participants stratified by scar formation type.^a^

Characteristics	Overall (*n* = 991)	Mature scar (*n* = 337)	Abnormal scar	
			Hypertrophic scar (*n* = 639)	Keloid (*n* = 15)
Age, years	34.2 ± 4.6	34.0 ± 4.7	34.3 ± 4.6	35.3 ± 4.0
Gestational week	38.71 ± 1.78	38.76 ± 1.63	38.68 ± 1.82	38.47 ± 3.14
Parity	1.56 ± 0.71	1.58 ± 0.71	1.54 ± 0.71	1.60 ± 0.63
Primary or repeat CS				
Primary CS	674 (68%)	217 (64%)	447 (70%)	10 (67%)
Repeat CS	317 (32%)	120 (36%)	192 (30%)	5 (33%)
Emergency				
Emergency	505 (51%)	162 (48%)	332 (52%)	11 (73%)
Non‐emergency	486 (49%)	175 (52%)	307 (48%)	4 (27%)
Use of skin stapler	230 (23%)	70 (21%)	155 (24%)	5 (33%)
Incision				
Vertical	333 (34%)	101 (30%)	220 (34%)	12 (80%)
Transverse	658 (66%)	236 (70%)	419 (66%)	3 (20%)
Complications				
Hypertension	125 (13%)	42 (12%)	81 (13%)	2 (13%)
DM	9 (0.9%)	4 (1.2%)	5 (0.8%)	0 (0%)
GDM	64 (6.5%)	27 (8.0%)	37 (5.8%)	0 (0%)
Atopic dermatitis	33 (3.3%)	19 (5.6%)	14 (2.2%)	0 (0%)
AID	16 (1.6%)	4 (1.2%)	12 (1.9%)	0 (0%)

Abbreviations: AID, autoimmune disease, CS, cesarean section; DM, diabetes mellitus; GDM, gestational diabetes mellitus.

^a^ Data are presented as mean ± standard deviation or as number (percentage).

**FIGURE 2:**
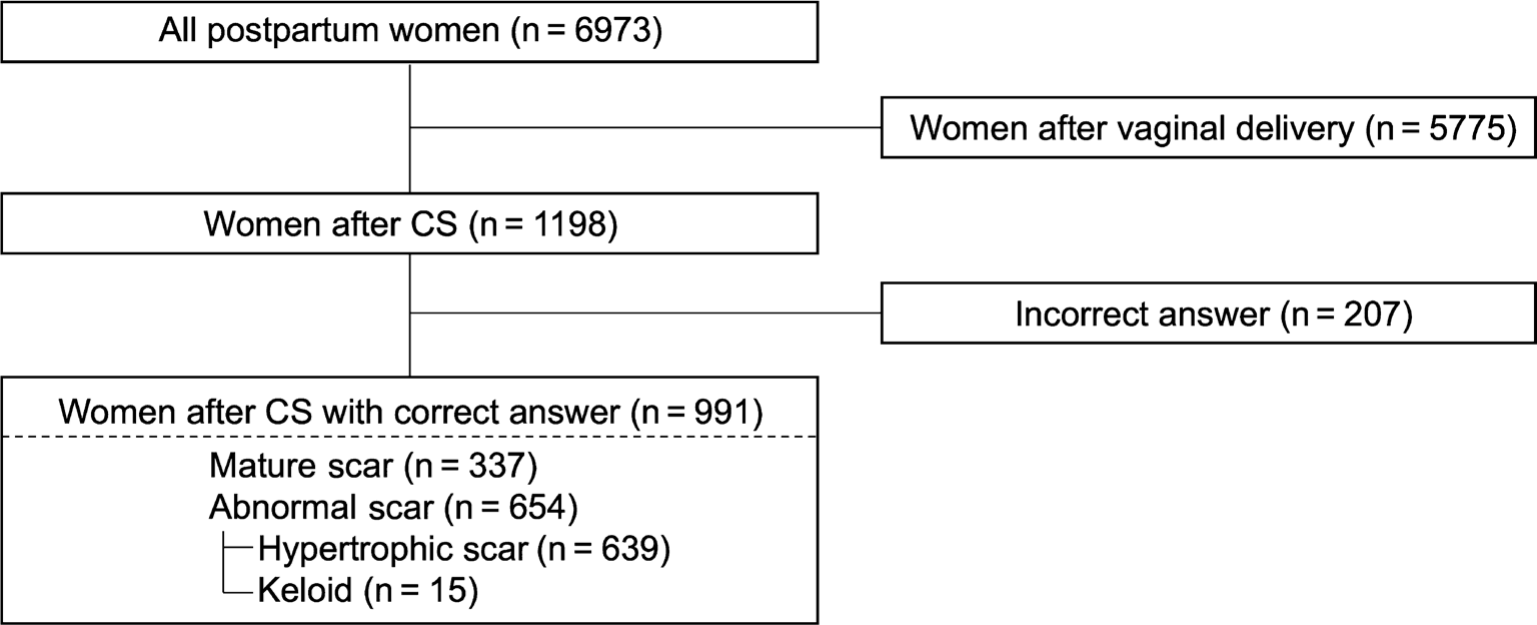
Participant flow diagram showing the selection process of study participants. Among 6973 postpartum women who responded to the screening survey, 1198 had undergone cesarean section. After excluding 207 responses with incorrect answers, 991 women were included in the final analysis: 337 with mature scars, 639 with hypertrophic scars, and 15 with keloids.

Analysis of scar formation patterns among the 991 participants revealed that 337 (34.0%) developed mature scars, but 654 (66.0%) presented with abnormal scars. Among those with abnormal scars, 639 (64.4%) had hypertrophic scars and 15 (1.6%) developed keloids (Figure 2).

**FIGURE 3:**
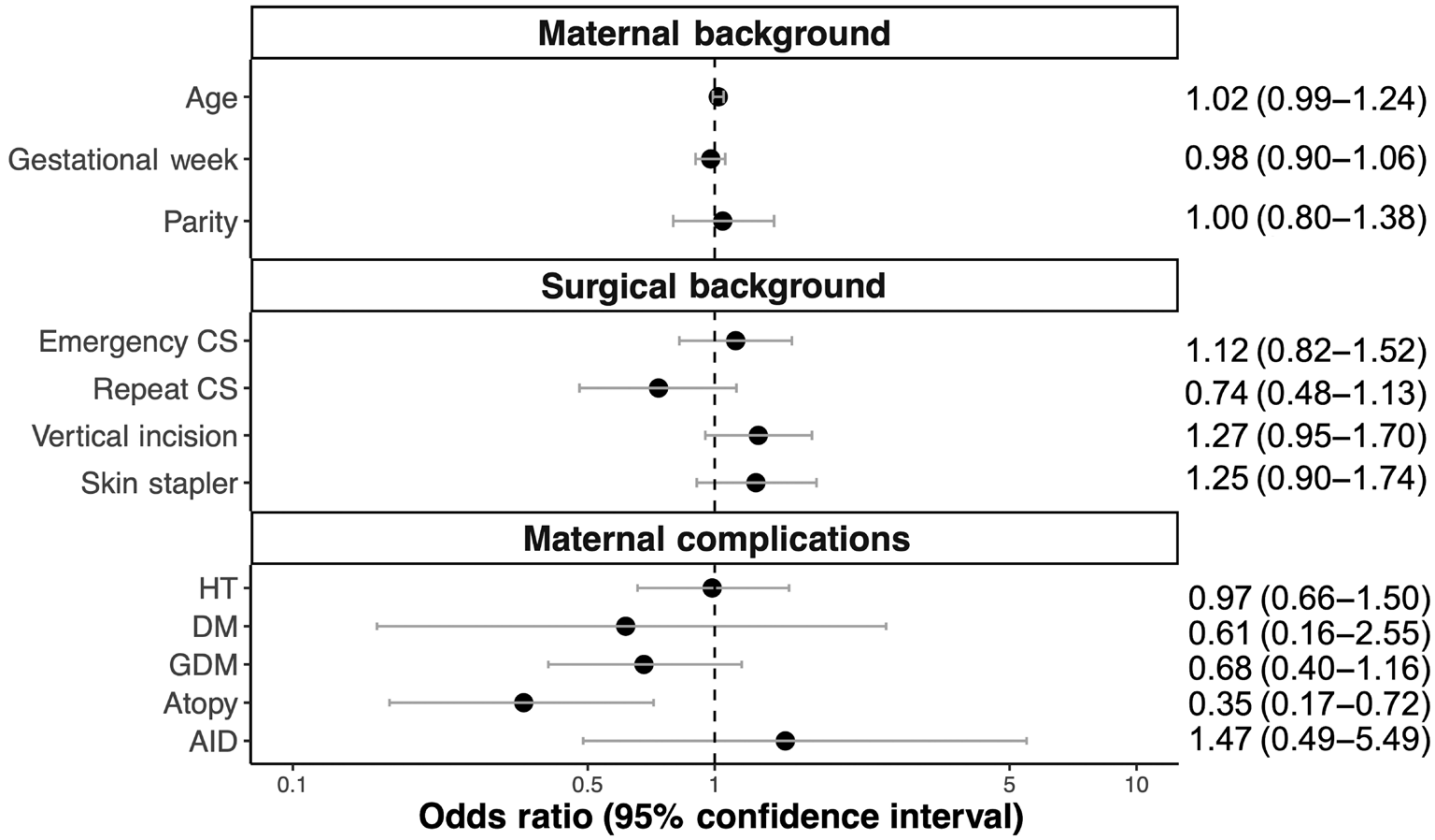
Forest plot of risk factors for abnormal scar formation. Odds ratios and 95% confidence intervals for potential risk factors associated with abnormal scar formation after cesarean section, based on multiple logistic regression analysis. Factors are grouped into maternal background, surgical background, and maternal complications. Atopic dermatitis showed a significant protective effect (*P* < 0.05). AID, autoimmune disease; DM, diabetes mellitus; GDM, gestational diabetes mellitus; HT, hypertension.

Multivariate logistic regression analysis identified atopic dermatitis as having a protective effect against abnormal scar formation (odds ratio [OR] 0.35, 95% confidence interval [CI] 0.17–0.72). Several factors showed trending associations with increased risk of abnormal scar formation, including repeat CS (OR 0.74, 95% CI 0.48–1.13), vertical incision (OR 1.27, 95% CI 0.96–1.70), and skin stapler use (OR 1.25, 95% CI 0.90–1.74), although these did not reach statistical significance (Figure 3).

**FIGURE 4:**
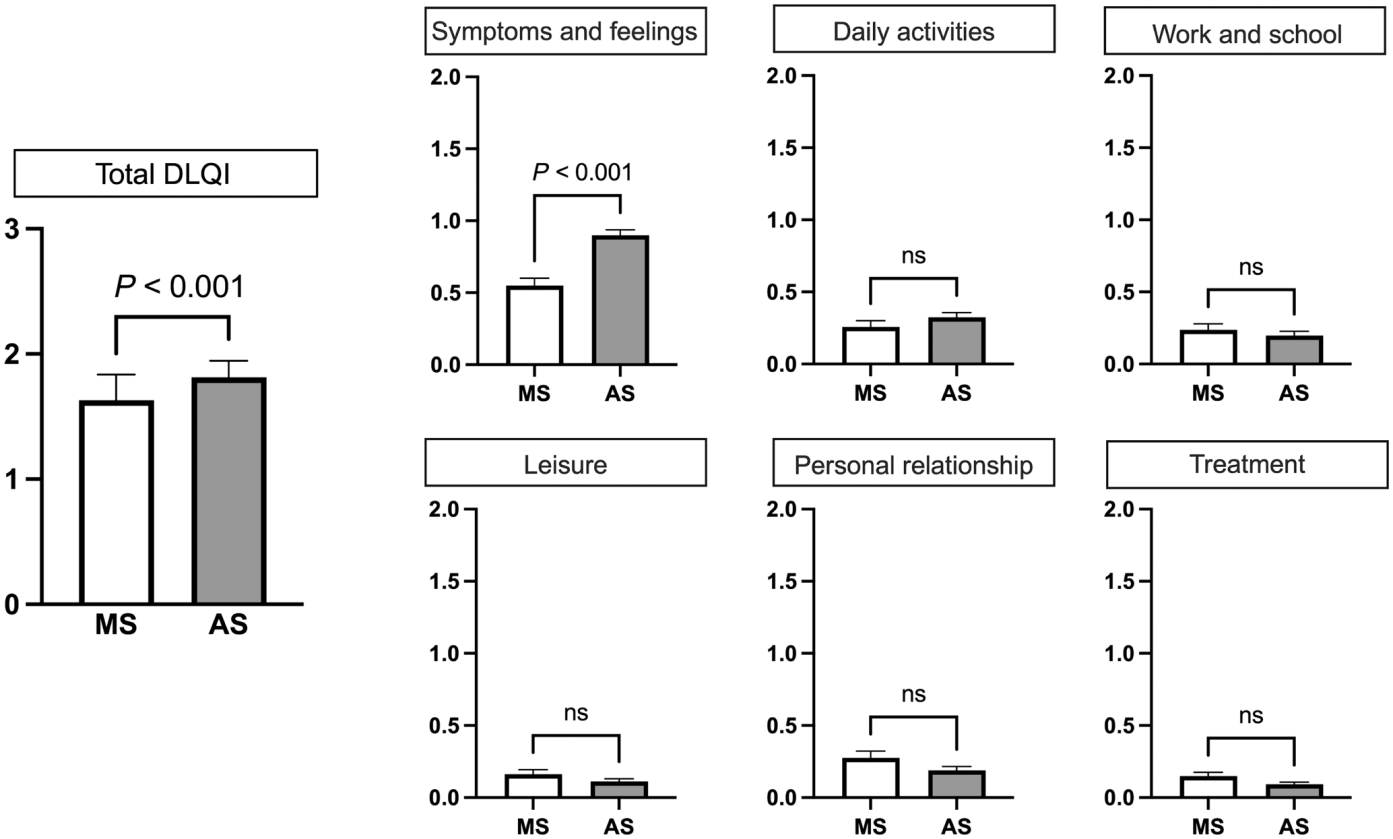
Comparison of Dermatology Life Quality Index (DLQI) scores between mature and abnormal scar groups. Bar graphs showing total DLQI scores and domain‐specific subscores between mature scar (MS) and abnormal scar (AS) groups. The abnormal scar group showed significantly higher total DLQI scores and symptoms and feelings subscores (*P* < 0.001), whereas other domains showed no statistically significant differences (ns, not significant). Error bars represent standard error of the mean.

The DLQI analysis demonstrated an overall mean score of 1.85, indicating minimal QoL impairment in the study population, falling within the “small effect” range according to established interpretation guidelines. The abnormal scar group showed significantly higher scores, indicating worse QoL (median 1, interquartile range [IQR] 0–2) compared with the mature scar group (median 0, IQR 0–1; *P* < 0.001). Domain‐specific analysis revealed the greatest impairment in the symptoms and emotions domain (*P* < 0.001), followed by daily activities (*P* < 0.01) (Figure 4).

**FIGURE 5:**
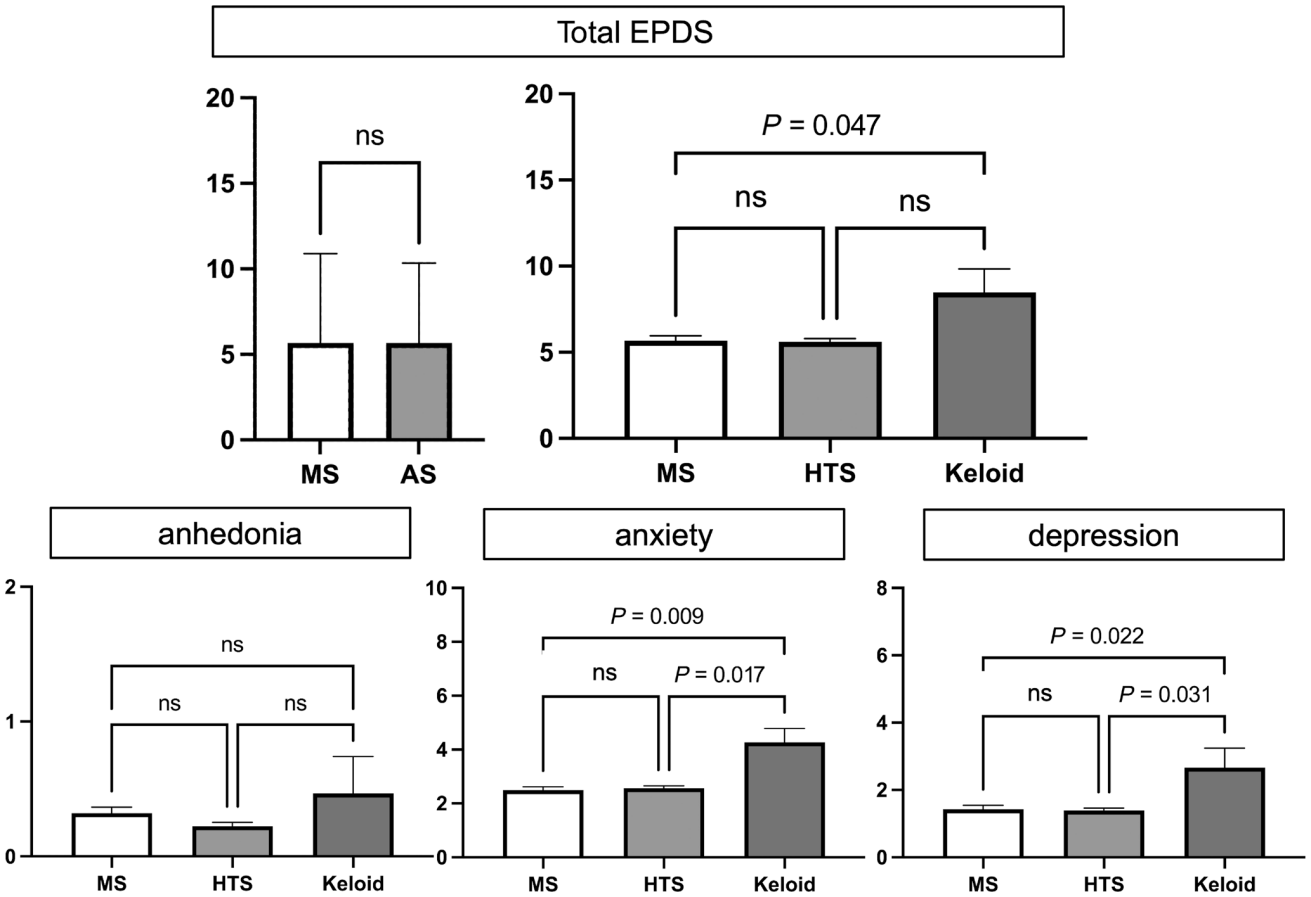
Comparison of Edinburgh Postnatal Depression Scale (EPDS) scores. Upper panel shows total EPDS scores comparing mature scar (MS), hypertrophic scar (HTS), and keloid groups. Lower panels show subscale scores for anhedonia, anxiety, and depression. The keloid group demonstrated statistically significant differences in anxiety (*P* < 0.01) and depression (*P* < 0.05) subscales compared with the mature scar group, while other comparisons were not statistically significant (ns, not significant). Error bars represent standard error of the mean.

Assessment of mental health outcomes using EPDS revealed a mean score of 5.68, indicating mild depression risk in the overall population and falling below the clinical threshold. However, 23.7% of participants exceeded the clinical threshold score of 9, indicating clinically significant depression risk. Notably, the keloid subgroup demonstrated significantly higher total scores compared with those with mature scars, with particular elevation in anxiety and depression subscales (Figure 5). This finding suggests a potential dose–response relationship between scar severity and mental health impairment.

## 4 DISCUSSION

Our analysis revealed three key findings. First, the prevalence of abnormal scar formation (66.0%) following CS substantially exceeded previous estimates of 31%–41%.^11^, ^12^ Second, women with abnormal scars experienced significant QoL impairment, particularly in the symptoms and emotions domain. Third, we found that women with keloid formation had significantly higher EPDS scores, with particular elevation in anxiety and depression subscales, indicating a greater risk of PPD.

The prevalence of abnormal scars in our study substantially exceeds previously reported rates, suggesting potential under‐recognition of this complication in clinical practice. This difference may reflect several factors: (1) our large sample size providing more accurate prevalence estimates compared with previous studies typically involving fewer than 100 participants, (2) the use of patient‐reported outcomes, which may be more sensitive to subjective scar characteristics, (3) potential population‐specific factors in Japanese women, and (4) possible underreporting in smaller previous studies due to selection bias. The higher prevalence emphasizes the clinical significance of this under‐recognized complication and supports the need for routine postpartum scar assessment. Specifically, our finding of 66.0% prevalence of abnormal scarring significantly exceeds previously reported rates of 31%–41%.^11^, ^12^ This difference is noteworthy considering the global paucity of literature on this topic. Previous studies have been constrained by small sample sizes, typically involving fewer than 100 participants, which may have led to underestimation of the true prevalence of this complication worldwide. Our study provides the first large‐scale, nationwide assessment of this clinically significant issue. Our findings regarding QoL impairment align with previous studies of hypertrophic scars and keloids in other contexts.^6^, ^7^, ^8^ The identification of a potential link between keloid formation and PPD provides a crucial new dimension to our understanding of maternal mental health. The unexpected protective effect of atopic dermatitis against abnormal scar formation warrants further investigation and may provide insights into mechanisms of aberrant wound healing.

Our findings emphasize the need for enhanced perioperative scar prevention protocols in CS procedures. Regular post‐CS scar assessment should be integrated into routine postpartum care. Women who developed keloids showed worse mental health outcomes, highlighting the need for integrated mental health screening and care, particularly for those with abnormal scars. Early identification of high‐risk patients could enable timely intervention and potentially prevent adverse outcomes.

These results highlight several critical areas for future investigation. Prospective studies are needed to evaluate the effectiveness of preventive interventions for abnormal scar formation. The mechanisms linking scarring to maternal mental health require detailed investigation, potentially offering new therapeutic targets. Long‐term maternal outcomes should be studied to understand the full impact of abnormal scarring. Additionally, the development of targeted therapeutic approaches, informed by our findings regarding risk factors and protective elements, represents an important direction for future research.

Our study's strengths include its large, geographically representative sample across all Japanese prefectures, the use of validated assessment tools for both scar evaluation and psychological outcomes, and comprehensive outcome evaluation yielding novel findings with immediate clinical relevance. However, several limitations should be considered. A major limitation of our study is the use of patient self‐assessment for scar classification rather than the reference standard physician evaluation recommended by the JSW Scar Scale 2015. This approach may have led to misclassification of scar types, as patients cannot assess crucial tactile characteristics such as scar thickness, firmness, texture, and sensation that are essential for accurate scar classification according to established clinical criteria. The absence of validation studies comparing photographic self‐assessment with clinical evaluation for post‐cesarean scars further limits the reliability of our scar classification findings. Although this methodologic compromise enabled large‐scale data collection across all Japanese prefectures, it represents a significant limitation that may affect the validity of our results. The cross‐sectional design limits our ability to establish causal relationships between abnormal scarring and mental health outcomes; longitudinal studies are needed to determine temporal relationships. Additionally, the internet‐based sampling method may have introduced selection bias toward more digitally literate participants, potentially affecting the generalizability of our findings to all postpartum women in Japan. Despite these limitations, our study provides the first large‐scale, nationwide assessment of this clinically significant issue with robust geographic representation across all Japanese prefectures.

In conclusion, the present study demonstrates three key findings regarding CS outcomes in Japan: abnormal scar formation is highly prevalent, abnormal scars impair maternal quality of life, and keloid formation is linked to an increased risk of postpartum depression.

## AUTHOR CONTRIBUTIONS

YS designed the study, collected and analyzed data, and wrote the original draft; HK contributed to conceptualization, supervision, and manuscript review; CK, DU, NE, SN, TO, TS, and YO contributed to methodology and project administration; RO provided resources and supervision; and YH provided supervision. All authors reviewed and approved the final manuscript.

## CONFLICT OF INTEREST STATEMENT

All authors have received honoraria from Johnson & Johnson K.K. for presentations at company‐sponsored or co‐sponsored seminars related to obstetrics and gynecology where the findings from this survey research were discussed. These financial relationships have been properly disclosed and managed in accordance with institutional policies.

## Data Availability

The data that support the findings of this study are not publicly available because of privacy restrictions and the original terms of participant consent, which did not include provisions for broader data sharing.
